# Reassessment of the function of somatolactin alpha in lipid metabolism using medaka mutant and transgenic strains

**DOI:** 10.1186/1471-2156-13-64

**Published:** 2012-07-24

**Authors:** Yuko Sasano, Asami Yoshimura, Shoji Fukamachi

**Affiliations:** 1 Laboratory of Evolutionary Genetics, Department of Chemical and Biological Sciences, Japan Women’s University, Mejirodai 2-8-1, Bunkyo-ku, Tokyo, 112-8681, Japan

**Keywords:** Somatolactin alpha (SLa), Medaka, Hepatic triglycerides, Pigmentation

## Abstract

**Background:**

Somatolactin alpha (SLa) is a fish-specific peptide hormone secreted from the pituitary. In medaka, SLa functions to darken the skin color and lack of SLa makes it pale. Transcription of *SLa* is enhanced or suppressed when fish are kept in dark or bright conditions, respectively, indicating SLa’s important role in background acclimation of the skin color. Bizarrely, however, the lack of SLa seems to cause the additional defect of increased triglycerides in organs, which could not be rescued (decreased) by its overexpression.

**Results:**

To assess this enigmatic result, we investigated genetic (the *SLa*, *Slc45a2*, *r*, and *Y* genes) and nongenetic (age, fasting, water temperature, and background color) effects on hepatic triglycerides. These experiments found that percent hepatic triglycerides quickly change in response to external/internal environments. Effects of *SLa* seemed to be much less obvious, although it may increase the proportion of hepatic triglycerides at least during certain breeding conditions or under certain genetic backgrounds.

**Conclusions:**

The present results do not exclude the possibility that SLa takes part in lipid metabolism or other physiological processes. However, we suggest that skin-color regulation is the only definite role of SLa so far demonstrated in this species.

## Background

Somatolactin alpha (SLa) is the closest relative of growth hormone (GH) secreted from the pars intermedia of the pituitary of “fish” species. For unknown reasons, tetrapods have secondarily lost the *SLa* gene during evolution after the lungfish branched off [1]. Although functions of SLa have been extensively studied using various species, results are often conflicting (see refs in [2]) and its physiological roles *in vivo* remain largely unclear.

Medaka (*Oryzias latipes*) is known as a powerful model for studying development and genetics [3], and there are many kinds of skin-color variants that have long been kept as pets in Japan [4]. One of the variants, *color interfere* (*ci*), is colored pale gray (instead of wild-type brown) because of abnormal proliferation and differentiation of pigment cells (chromatophores) in the skin. We previously identified that *ci* has a frame-shift mutation on the *SLa* gene [5] and could rescue the *ci* phenotype by transgenic overexpression of SLa [2]; i.e., the pale gray skin of *ci* became dark brown in the *SLa*-transgenic strain (Actb-SLa:GFP). Because both *ci* and Actb-SLa:GFP fish normally develop, grow, and reproduce as wild-type fish under ordinary breeding conditions, SLa seemed to play an essential role only in skin-color regulation. Indeed, it is well known that medaka, and many other fish species, acclimate their skin color to their surroundings (cryptic coloration [6]) and *SLa* is actually upregulated in dark conditions [5,7-9]. More recently, we found that the colors formed by SLa function as sexual traits in medaka (nuptial coloration [10]). These findings suggest that SLa plays important roles for successful survival and reproduction in nature via skin-color regulation.

A receptor for SLa (SLR) was identified in salmon [11] (but see [12]). Unexpectedly, its medaka orthologue was ubiquitously expressed in various organs with the highest level, not in the skin, but in the liver and muscles [13]. Hence, we expected that the pale *ci* and the dark Actb-SLa:GFP should have additional defects most likely in these organs. From this point of view, phenotypes of the cobalt variant of rainbow trout looked suggestive, because the fish lacks most of the pars intermedia of the pituitary, decreases SLa in the plasma, has pale skin (as *ci*), and is obese due to excessive fat accumulation in the abdominal cavity [14]. Furthermore, the physiological function of SLa in lipid metabolism has also been suggested in other species [11,15-17]. As expected, we detected more triglycerides in the liver and muscles of *ci* than we did in those of wild-type fish [13]. Strangely, however, this fat accumulation in *ci* could not be rescued in Actb-SLa:GFP, unlike the case for skin coloration [2]. Thus, the causal relationship between SLa and lipid metabolism remains an open question that should be carefully reassessed.

Generally, traits (phenotypes) of animals can be affected by genes (genotypes) and environments. In previous experiments, we used fish that were born and bred in a laboratory, believing that the different strains were under identical conditions and could be used to evaluate genetic effects on phenotypes. Water temperature, water quality, and light cycle were stably controlled by an automated water filtration and circulating system, and we fed fish using the same diets. However, the amount of diet, the speed of water flow into tanks, algae growing on tank walls, excrement remaining at the bottom, or other conditions may not be exactly identical between tanks, and such differences may significantly affect lipid metabolism. Thus, we hypothesized that the unrescued lipid phenotype of Actb-SLa:GFP [2] reflects not only genetic differences between the mutant/transgenic strains, but also such (seemingly subtle) environmental artifacts, which misled our conclusion regarding SLa’s function (hepatic triglycerides were actually shown to be sensitive to diet [18]). In this study, we raised various medaka strains under various breeding conditions and measured hepatic triglycerides to reassess the potential role of SLa in lipid metabolism.

## Results

### Increase of hepatic triglycerides during aging

As described in the Methods section, we first improved the protocol for lipid extraction from the liver and could successfully decrease the variance of the data (Table 1). Using this method, we measured hepatic triglycerides of the Actb-SLa:GFP strain twice after the improvement of the protocol. The second experiment (“After 2” in Table 1) was performed two months after the first experiment (“After 1”) using fish that had been kept in the same tank under the same conditions. However, the amount of hepatic triglycerides per liver (percent hepatic triglycerides; w/w) was strikingly increased in the second experiment (*P* < 0.001, unpaired two-tailed *t* test). We did not detect obvious deterioration of water quality or infectious disease during the two months. The only differences between the first and second experiments that we could think of were fish age and rearing density in the tank. To investigate reproducibility of this result, we performed the following experiment.

**Table 1:** Changes in variance after improvement of the experimental protocol

**Strains**	**Protocol improvement**	**n**	**Proportion of hepatic triglycerides**			**Coefficient of variation**
			**Mean**	**Max**	**Min**	
Hd-rr	Before	24	2.58	7.90	0.51	0.63
	After	16	0.51	0.80	0.20	0.30
*ci*	Before	20	3.44	8.64	0.46	0.63
	After	19	0.76	1.86	0.45	0.47
Actb-SLa:GFP	Before	9	2.86	4.84	0.30	0.53
	After 1^*^	5	0.50	0.70	0.38	0.26
	After 2^*^	13	2.26	4.37	0.37	0.26

*: Data from younger (After 1) and older (After 2) fish (see text).

**Figure 1:**
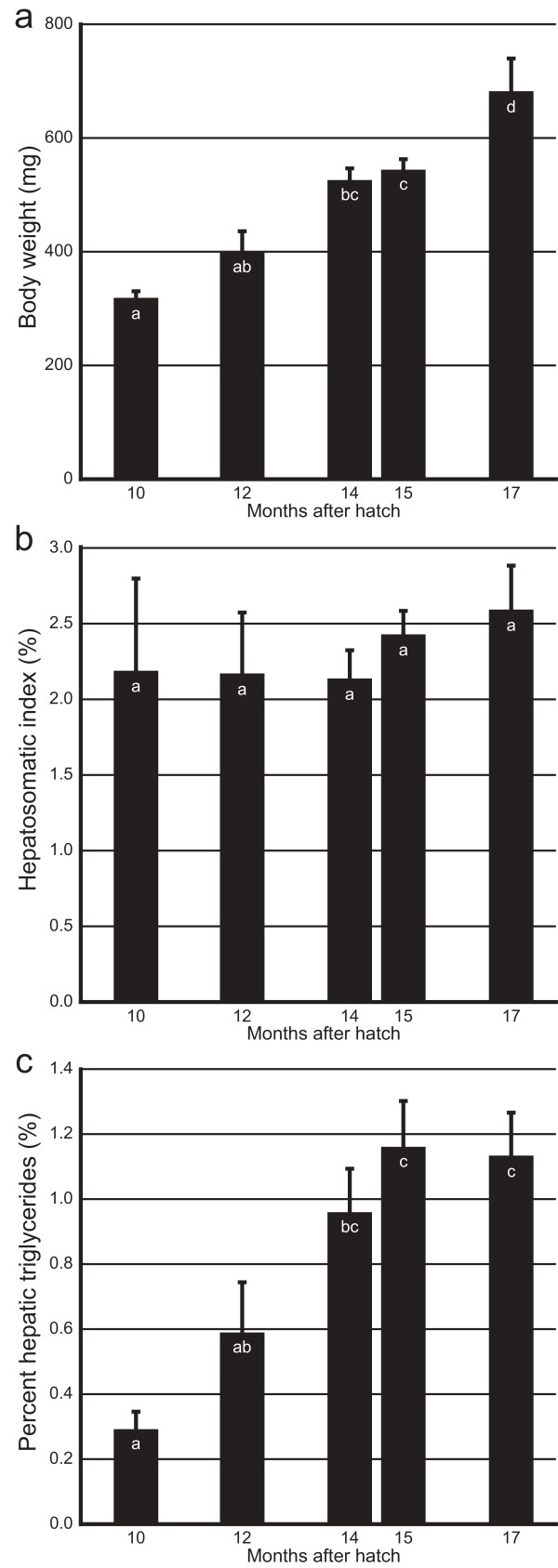
**Effects of aging, rearing density, or other breeding conditions on hepatic triglycerides in *O. celebensis*.** Mean and SEM of body weight (**a**), HSI (**b**), and percent hepatic triglycerides (**c**) are shown; *n* = 6, 6, 8, 8, and 5 for 10, 12, 14, 15, and 17 months after hatch, respectively. Letters in the bars indicate significant differences between the groups (*P* < 0.05, one-way ANOVA and Tukey’s *post hoc* HSD test).

We had a relatively large stock of fully matured *O. celebensis* that were born in the same week. Five times in eight months, we randomly chose five to eight fish from the stock and measured their hepatic triglycerides. These stockfish were still growing and increased their body weight during the eight months (Figure1a). Whereas the relative weight of the liver to the body (hepatosomatic index; HSI) did not change (Figure1b), percent hepatic triglycerides were steeply increased and significant increase could be detected within three months (Figure1c). Thus, fish age, rearing density, and/or other environmental changes that may occur in this experimental system seem to have nonnegligible effects in lipid metabolism. Given that the proportion of hepatic triglycerides greatly varies, even within the same strain, it seemed necessary to equalize both the external and the internal environments of fish more strictly than we had done in previous experiments to evaluate genetic effects. In the following experiments, we prepared fish that were born in the same week, provided uniform breeding conditions as far as possible (including tank size, fish density, water filtration, removal of algae on tank walls), and measured their hepatic triglycerides.

### Effects of overwintering on hepatic triglycerides

Medaka can live at various water temperatures; adult fish can survive snowy winters in the field [19]. We put fish of the same age into two tanks of the same size (50 × 30 × 29 cm) in the same density (22 fish/tank). For eight months (from July 2011 to February 2012), one of the tanks was kept in the laboratory (27°C) and the other was placed outdoors. The water temperature of the outside tank dropped to 4°C during this period and some fish died (as occurred in the inside tank) before we measured their hepatic triglycerides. The survivors were 10 *ci* (plus two OK-cab *Ci*/*Ci*; *SLa*^*–*^/*SLa*^*–*^; *B*′/*B*′; *R*/*R*) in the inside tank, and six *ci* (plus six OK-cab) in the outside tank. Because we could obtain only two OK-cab from inside and fish age is the same only within, but not between the strains, only data from *ci* were used for the following comparisons (note that fish densities were equal between the inside and outside tanks: 12 fish per tank).

As shown in Table 2, body weight did not differ between the groups (*P* = 0.109). Liver weight was significantly increased in the fish kept outside, and this hepatic hypertrophy could also be detected as a significant increase in the HSI. As a result, the fish kept outside stored more hepatic triglycerides than the fish kept inside, but percent hepatic triglycerides did not differ between the groups (*P* = 0.155). Therefore, medaka seemed to store triglycerides for overwintering by increasing the entire volume of the liver, instead of increasing the proportion of triglycerides. In addition, this result shows that this hepatic hypertrophy could occur independently of SLa, because *ci* does not express any functional SLa [5]. 

**Table 2:** **Effects of overwintering on hepatic triglycerides in*ci*(mean±SEM)**

**Tank**	**Sex**	**n**	**Body weight (g)**	**Liver weight (mg)**	**HSI (%)**	**Hepatic triglycerides**	
						**Total (ng)**	**Percentage (%)**
Inside	Male	6	0.37±0.02	11.5±1.0	3.1±0.2	39±5	0.34±0.02
	Female	4					
Outside	Male	3	0.42±0.03	25.8±2.1*	6.3±0.07*	115±18*	0.45±0.07
	Female	3					

*: Significant increase in the outside fish (*P* < 0.05, two-tailed multiple *t* test with Bonferroni correction).

### Effects of fasting, background color, and SLa expression on hepatic triglycerides

Next, we prepared four tanks of the same size (24 × 17 × 12 cm) in which six adult fish (two each [a male and a female] of *ci*, Actb-SLa:GFP, and HNI) were bred in the laboratory. All fish were born in the same week. One tank was kept under ordinary breeding conditions. Two tanks were kept under the same conditions, except that the tank walls were painted black or white. The last tank was not painted, but no food was given to the inhabitants. After one month, no fish had died and we used all 24 fish for measuring percent hepatic triglycerides.

**Figure 2:**
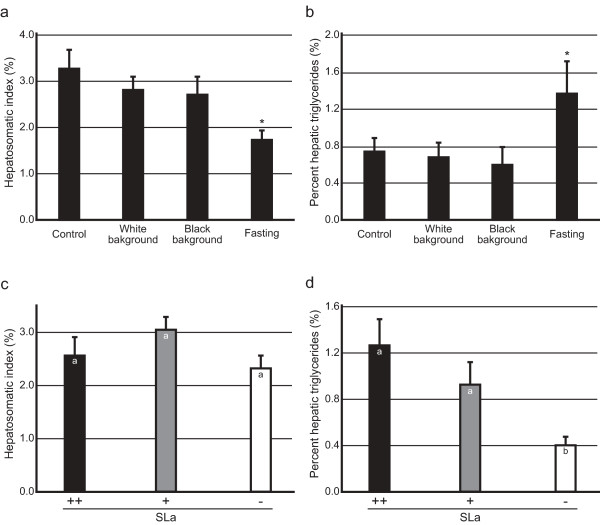
**Effects of background colors, fasting, and SLa expression on hepatic triglycerides.** Four pairs of three strains (*ci*, Actb-SLa:GFP, and HNI) were kept under four breeding conditions (control, white background, black background, or fasting). **a** and **b**, environmental effects on the HSI (**a**) and percent hepatic triglycerides (**b**). **c** and **d**, genetic effects on the HSI (**c**) and percent hepatic triglycerides (**d**). Letters in the bars indicate significant differences between the groups according to a two-way ANOVA and a Dunnett *post hoc* (**a** and **b**) or Tukey’s *post hoc* HSD (**c** and **d**) test.

No interaction between the environmental and genetic conditions could be detected using a two-way ANOVA in body weight (*P* = 0.936), liver weight (*P* = 0.976), HSI (*P* = 0.947), hepatic triglycerides (*P* = 0.987), or percent hepatic triglycerides (*P* = 0.088). In terms of the environmental comparison, a *post hoc* Dunnett test detected a significant decrease in the HSI in the fasted fish (*P* = 0.024; Figure2a). Interestingly, however, percent hepatic triglycerides of the fasted fish were significantly higher than in the control group (*P* = 0.015; Figure2b). Neither black nor white background seemed to affect the HSI or percent hepatic triglycerides. Considering that the amount of hepatic triglycerides did not differ between the four groups (*P* = 0.670; data not shown), the atrophied and fatty liver of the fasted fish would be because of consumption of stored hepatic nutrients other than triglycerides (e.g., carbohydrates) during the fasting period.

**Figure 3:**
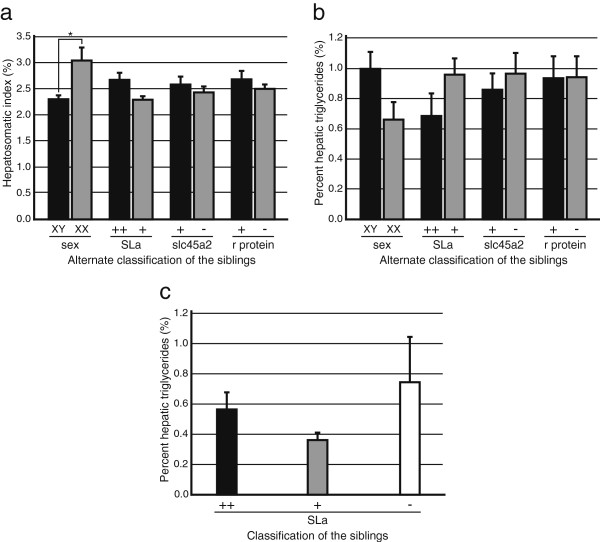
**Effects of the*Y*, *ci*, *SLa*, *b*, and *r *genes on hepatic triglycerides. a**, HSI of the Cross I siblings. Asterisks indicate significant differences (*P < 0.05; two-tailed multiple *t* test with Bonferroni correction). **b**, percent hepatic triglycerides of the Cross I siblings. **c**, percent hepatic triglycerides of the Cross II siblings.

In terms of the genetic comparison, Tukey’s *post hoc* HSD test detected no significant difference between the *ci*, Actb-SLa:GFP, and HNI strains (*P* = 0.255; Figure2c). Percent hepatic triglycerides, on the other hand, were significantly lower in *ci* in comparison with those of HNI (*P* = 0.018) or Actb-SLa:GFP (*P* < 0.001; Figure2d). Since the genomic difference between Actb-SLa:GFP and *ci* is only the presence or absence of the *SLa* transgene, SLa seems to function to increase percent hepatic triglycerides without increasing the entire weight of the liver (but see Figure3c).

### Effects of the *ci*, *SLa*, *b*, *r*, and *Y* loci on hepatic triglycerides

We further investigated the potential role of SLa to regulate hepatic triglycerides. For this purpose, we performed two crosses (Crosses I and II; see Methods) and prepared F_2_ fish with various genotypes for the endogenous *ci*, *b*, *r*, *Y* loci and the exogenous *SLa* locus, but with similar genomic backgrounds (Table 3). These F_2_ siblings were born in the same week and bred in the same tank. After they reached sexual maturity, we phenotyped/genotyped the fish (see Methods) and measured their hepatic triglycerides.

**Table 3:** **Classification of the F**_**2**_**siblings obtained from Crosses I and II**

**Genotypes of the Cross I siblings**	Phenotypes of the siblings*
[*Ci*/*Ci; B*/*b*; *R*/*r*; *SLa*^*+*^/*SLa*^*–*^], [*Ci*/*ci; B*/*b*; *R*/*r*; *SLa*^*+*^/*SLa*^*–*^]	black, orange, SLa++
[*Ci*/*Ci; B*/*b*; *R*/*r*; *SLa*^*–*^/*SLa*^*–*^], [*Ci*/*ci; B*/*b*; *R*/*r*; *SLa*^*–*^/*SLa*^*–*^]	black, orange, SLa+
[*Ci*/*Ci; B*/*b*; *r*/*r*; *SLa*^*+*^/*SLa*^*–*^], [*Ci*/*ci; B*/*b*; *r*/*r*; *SLa*^*+*^/*SLa*^*–*^]	black, SLa++
[*Ci*/*Ci; B*/*b*; *r*/*r*; *SLa*^*–*^/*SLa*^*–*^], [*Ci*/*ci; B*/*b*; *r*/*r*; *SLa*^*–*^/*SLa*^*–*^]	black, SLa+
[*Ci*/*Ci; b*/*b*; *R*/*r*; *SLa*^*+*^/*SLa*^*–*^], [*Ci*/*ci; b*/*b*; *R*/*r*; *SLa*^*+*^/*SLa*^*–*^]	orange, SLa++
[*Ci*/*Ci; b*/*b*; *R*/*r*; *SLa*^*–*^/*SLa*^*–*^], [*Ci*/*ci; b*/*b*; *R*/*r*; *SLa*^*–*^/*SLa*^*–*^]	orange, SLa+
[*Ci*/*Ci; b*/*b*; *r*/*r*; *SLa*^*+*^/*SLa*^*–*^], [*Ci*/*ci; b*/*b*; *r*/*r*; *SLa*^*+*^/*SLa*^*–*^]	SLa++
[*Ci*/*Ci; b*/*b*; *r*/*r*; *SLa*^*–*^/*SLa*^*–*^], [*Ci*/*ci; b*/*b*; *r*/*r*; *SLa*^*–*^/*SLa*^*–*^]	SLa+
**Genotypes of the Cross II siblings**	Phenotypes of the siblings*
[*Ci*/*ci; B*/*B*; *R*/*R*; *SLa*^*+*^/*SLa*^*–*^], [*Ci*/*ci; B*/*B*; *R*/*r*; *SLa*^*+*^/*SLa*^*–*^],	black, orange, SLa++
[*Ci*/*ci; B*/*b*; *R*/*R*; *SLa*^*+*^/*SLa*^*–*^], [*Ci*/*ci; B*/*b*; *R*/*r*; *SLa*^*+*^/*SLa*^*–*^],	
[*ci*/*ci; B*/*B*; *R*/*R*; *SLa*^*+*^/*SLa*^*–*^], [*ci*/*ci; B*/*B*; *R*/*r*; *SLa*^*+*^/*SLa*^*–*^],	
[*ci*/*ci; B*/*b*; *R*/*R*; *SLa*^*+*^/*SLa*^*–*^], [*ci*/*ci; B*/*b*; *R*/*r*; *SLa*^*+*^/*SLa*^*–*^]	
[*Ci*/*ci; B*/*B*; *R*/*R*; *SLa*^*–*^/*SLa*^*–*^], [*Ci*/*ci; B*/*B*; *R*/*r*; *SLa*^*–*^/*SLa*^*–*^],	black, orange, SLa+
[*Ci*/*ci; B*/*b*; *R*/*R*; *SLa*^*–*^/*SLa*^*–*^], [*Ci*/*ci; B*/*b*; *R*/*r*; *SLa*^*–*^/*SLa*^*–*^]	
[*ci*/*ci; B*/*B*; *R*/*R*; *SLa*^*–*^/*SLa*^*–*^], [*ci*/*ci; B*/*B*; *R*/*r*; *SLa*^*–*^/*SLa*^*–*^],	black, orange, SLa^*–*^
[*ci*/*ci; B*/*b*; *R*/*R*; *SLa*^*–*^/*SLa*^*–*^], [*ci*/*ci; B*/*b*; *R*/*r*; *SLa*^*–*^/*SLa*^*–*^]	

*: SLa++ overexpression, SLa+ normal expression, SLa^*–*^ no expression. SLa expression from the wild-type *Ci* allele is negligible when the exogenous *SLa* gene is present.

We collected 78 F_2_ fish from Cross I (Table 4) and completed the measurement of hepatic triglycerides within two weeks to minimize the effect of aging or density change (see Figure1). Neither the *b* nor the *r* locus appeared to have a significant effect on the HSI or percent hepatic triglycerides (Figures 3a and 3b). In terms of the *Y* locus, the HSI (but not percent hepatic triglycerides) was significantly higher in females (3.1± 0.2%; mean±SEM) than in males (2.3±0.1%; *P* < 0.05; Figure3a). We found that this had been the case in the data in Figure2 (2.2±0.2% in males and 3.1±0.3% in females [n = 12 for each]; *P* = 0.015, two-tailed *t* test). The result was also reproduced in an independent experiment using Hd-rr (2.3±0.1% in males [n = 8] and 3.6±0.2% in females [n = 5]), but not in experiments using *ci* (3.1±0.3% in males [n = 4] and 3.9±0.2% in females [n = 7]) or Actb-SLa:GFP (3.8±0.1% in males [n = 4] and 3.4±0.3% in females [n = 9]) (*P* < 0.05; two-tailed multiple *t* test with Bonferroni correction). Thus, medaka females tended to have a larger liver than males, but only under limited conditions (external/internal environments or genomic backgrounds). In terms of the *SLa* locus, neither the HSI nor percent hepatic triglycerides were significantly affected. This result is consistent with the results in Figures 2c and 2d showing no significant difference in the HSI or percent hepatic triglycerides between Actb-SLa:GFP (SLa++) and HNI (SLa+).

**Table 4:** **The number of F**_**2**_**siblings obtained from Cross I**

**Criteria of classification**		**Present**	**Absent**	**Not determined**	**Total**
*Y* gene		55 (male)	23 (female)	0	78
*SLa* transgene	male	19	32	5	78
female	12	8	2		
*B* allele	male	32	23	0	78
female	15	8	0		
*R* allele	male	25	19	11	78
female		12	8	3		

From Cross II, we obtained 30 adult fish (Table 5) and collected data within two days. This cross could produce three types of F_2_ siblings that express SLa at different levels: overexpression (SLa++), wild-type expression (SLa+), and no expression (SLa–). Although we did not measure body weight and therefore could not calculate the HSI in this experiment, the liver of females (3.9±0.2 mg) was significantly heavier than that of males (2.8±0.1 mg; *P* < 0.001), which was consistent with the results in Cross I. In terms of percent hepatic triglycerides, a significant difference could not be detected between the SLa++, SLa+, and SLa– F_2_ siblings (*F*_(2, 27)_ = 1.319, *P* = 0.284; one-way ANOVA; Figure3c). This result is, however, inconsistent with the result in Figure2d, which shows a significant difference between Actb-SLa:GFP (SLa++) and *ci* (SLa–). We discuss potential causes for this below.

**Table 5:** **The number of F**_**2**_**siblings obtained from Cross II**

**SLa expression**	**Excess**	**Normal**	**None**	**Total**
Male	10	6	5	30
Female	5	3	1	

## Discussion

### Advantages and disadvantages in medaka physiology

Two important facts, which we overlooked in previous experiments [2,13], became obvious with the present series of experiments: (1) the proportion of hepatic triglycerides rather sharply changes in association with environmental changes (Figure1c); and (2) microscopic trimming of the liver is necessary to avoid contamination of the oil-like droplets from the body cavity (Table 1). In the previous experiments, we used fish that had been kept under identical breeding conditions for at least three months, assuming that this step would minimize environmental effects on lipid phenotypes as it actually does in terms of skin-color phenotypes (background acclimation). However, the fish were about 0.5- to 1-year-old laboratory brood stock and their precise ages were unknown (because generations of lab strains are not always synchronically alternated). Moreover, we did not trim the liver before extracting lipids in the previous experiments. Therefore, we would have to admit that the significant increase in percent hepatic triglycerides in *ci*[13] may only reflect experimental artifacts. If this is the case, the phenotypes of Actb-SLa:GFP showing rescued skin color but unrescued hepatic triglycerides [2] would not be considered enigmatic. Regardless whether SLa may or may not control hepatic triglycerides, its effect is subtle compared with that on skin-color regulation and could easily be obscured by environmental conditions.

Although we successfully improved the stability of the data by the present protocol (Table 1), it may still not be perfect and needs to be further improved. The livers of medaka are so small (sometimes less than 10 mg) that even a tiny contamination of the oil-like droplets may markedly increase the amount of triglycerides. Another potential problem in the present protocol is that the liver of aged medaka (e.g., 17 months after hatch in Figure1) is so fragile that it partially breaks up during the trimming. This would not affect the proportion of hepatic triglycerides, but would reduce liver weight (and thereby the HSI) and the amount of hepatic triglycerides. Thus, the small body size of medaka would not provide the best model for this kind of physiological experiments. Nonetheless, we believe that we could obtain reliable data by analyzing a large number of samples (over 300 fish in total), which is one of the advantages of using this model organism for experiments.

### Potential function of medaka SLa to “increase” percent hepatic triglycerides

A role of SLa in fasting (lipid usage/storage) has long been suggested; however, results between species (or even researchers) are controversial. For example, increase of SLa during fasting was reported from gilthead sea bream [16,17] and European sea bass [15], which would indicate the “antiobese” effect of SLa. In contrast, its decrease during fasting was also reported from gilthead sea bream [20] and tilapia [21]. Moreover, no change was reported from rainbow trout [22,23] and rabbitfish [24]. Thus, the causal relationship between SLa and lipid metabolism remains generally ambiguous. Our study indicated that SLa has little (if any) role for winter fasting, because *ci* (with no functional SLa) did survive a snowy winter, as did the wild-type OK-Cab fish (Table 2). Genes other than *SLa* must be involved in this hepatic hypertrophy, even though up- and downregulation of SLa may occur during this period as reported from other fish species.

Our study also indicated that medaka SLa increases percent hepatic triglycerides (Figure2d). It should be noted that this change in the liver would be fundamentally different from hepatic hypertrophy during overwintering where only the liver weight, but not percent hepatic triglycerides, was increased (Table 2). This function of SLa, however, could not be reproduced in another experiment using different strains (Figure3). The reason for this inconsistency remains unknown, but effects of SLa on percent hepatic triglycerides may only be detectable at a certain growth stage (Figure1c) or under a certain genomic background (detectable under 100% *ci* background, but undetectable under the mixed F_2_ [*ci*:Hd-rr = 3:1] background). Alternatively, we may have overlooked the oil-like droplets during the liver trimming, which seems to be reflected as the increased variance (and mean) of the SLa– siblings in Figure3c. In any case, considering that *ci* survived a snowy winter (Table 2) and both *ci* and Actb-SLa:GFP could survive the complete fasting of one month as the wild-type HNI (Figures 2a and 2b), medaka SLa appears to have only a dispensable role in lipid metabolism during overwintering or fasting.

### A primary function of medakaSLa

As discussed above, the contribution of SLa to lipid metabolism remains unclear both in medaka and in other species. On the other hand, evidence supporting the relationship between SLa and skin pigmentation has been accumulating recently. Consistent with the case in medaka, SLa is upregulated under dark conditions in red drum [8] and cichlid [7,9] (but not in rainbow trout [22]). Melanin-concentrating hormone, which makes the skin color pale [25], suppresses SL release in goldfish [26] (although the opposite is the case in male cichlid [27]). Stripe formation in the skin was disrupted by ectopic (but mosaic) expression of *SLa* in zebrafish [28]. As listed, a concrete consensus about the SLa’s function in chromatophore regulation has not been obtained among species. We assume that this may reflect the fact that skin color is one of the most divergent traits of animals; i.e., functions of skin-color genes, including *SLa*, could be different between species.

We recently found that the color controlled by SLa is recognized as a sexual trait in medaka, suggesting SLa’s role in nuptial coloration [10]. From this standpoint, it is worth noting that the upregulation of SLa during reproduction or its downregulation after ovulation have been reported in many fish species [29-32]. That is, SLa would be upregulated not for reproduction itself (e.g., gonad/gamete maturation), but for attracting mating partners. It is well known that in species such as stickleback, guppy, and salmon, male fish often adopt “warm” (yellow-orange-red) colors for this purpose, a process that is enhanced by SLa in medaka [2]. However, it is not always the case and there are fish that prefer “cold” colors for mating [10,33]. The inconsistent results obtained in not only lipid metabolism but also skin-color regulation may be the result of this organismic diversity and the species-specific functions of SLa.

Our present results strongly suggest that the primary function of SLa in medaka is skin-color regulation. Besides the hepatic triglycerides, we previously suggested roles for SLa in regulating triglycerides in the muscles and cortisol in the plasma [13], which could also not be rescued by SLa overexpression [2]. Although we did not focus on these phenotypes in this study, these enigmatic results may also reflect similar experimental artifacts. If so, the upregulation of SLa on a black background [5,7-9] should be able to darken the skin color without unnecessarily increasing triglycerides in the organs or secreting stress hormones into the bloodstream.

The idea of investigating the liver and muscle phenotypes originally came from the fact that the SLR [11] is strongly expressed in these organs [13]. This receptor is, however, orthologous to the GH receptor (GHR) of tetrapods [1] and even counterevidence for its function (the nonbinding of SLa to the SLR) was recently reported [12]. Although the SLR was shown to be expressed in the skin and upregulated on a black background in cichlid [9], molecular mechanisms of SLa’s function on skin chromatophores may need to be reconsidered (e.g., another receptor, or the existence of second messengers). For this purpose, medaka provides powerful experimental opportunities where the gene-knockout method (TILLING) had been established [34]. If the SLR actually functions as a necessary and sufficient receptor for SLa, the phenotype of SLR-knockout medaka should resemble that of the SLa-deficient *ci*. In contrast, if the SLR functions only as a GHR, the knockout should exhibit a dwarf phenotype. We have already screened the knockout fish and are currently outcrossing to remove unrelated mutations in their genome (R. Komine and S. Fukamachi; unpublished). This experiment will provide conclusive evidence whether the SLR functions as a real receptor for SLa *in vivo*.

## Conclusion

Skin color is the only phenotype so far identified that SLa conspicuously affects in medaka. The causal relationship between SLa and lipid metabolism requires further investigation, but SLa should not play a constant and major role in regulating the proportion of hepatic triglycerides *in vivo* that could be measured by the present protocol. Further investigation with carefully designed experiments under various conditions is necessary to understand the entire aspect of SLa’s function.

## Methods

### Medaka strains

We focused on four loci (besides the sex-determining *Y* locus [35]) in this study. Those were the *ci* locus that expresses SLa, the *b* locus that expresses solute carrier family 45 member 2 (slc45a2) that is responsible for melanin synthesis [36], the *r* locus that is responsible for differentiation of xanthophores ([37]; its encoding protein has not been identified), and the transgenic *SLa* locus where the exogenous SLa-overexpressing construct [2] is integrated. We mainly used four strains: *ci*, Actb-SLa:GFP, wild-type HNI, and a double mutant of the *b* and *r* loci, Hd-rr (which is identical to the Hd-rR inbred strain except that its Y chromosome has the mutated *r* allele). Their genotypes could be described as follows: *cici*/*ci*; *SLa*^*–*^/*SLa*^*–*^; *B*/*B*; *R*/*R*, Actb-SLa:GFP *ci*/*ci*; *SLa*^*+*^/*SLa*^*+*^; *B*/*B*; *R*/*R*, HNI *Ci*/*Ci*; *SLa*^*–*^/*SLa*^*–*^; *B*/*B*; *R*/*R* and Hd-rr *Ci*/*Ci*; *SLa*^*–*^/*SLa*^*–*^; *b*/*b*; *r*/*r*. The *CiSLa*^*+*^*B*, and *R* alleles are dominant to the *ciSLa*^*–*^*b*, and *r* alleles, respectively.

All fish used in the present study were born and bred in laboratory until sexual maturity. After they matured, the fish were transferred into various conditions according to experimental designs (see Results). The water temperature was about 27°C and light was provided solely by ordinary fluorescent lamps for 14 hours per day. Fish were fed live *Artemia* and commercial flake food five times per day.

### Fish crossing and classification of F_2_ siblings

To obtain fish with various genotypes for the *ci*, *SLa*, *b*, and *r* loci, but with a reasonably uniform genomic background, we performed the following two crosses (female × male): Cross I: (Actb-SLa:GFP × Hd-rr) × Hd-rr, and Cross II: (Actb-SLa:GFP × Hd-rr) × *ci*. Because none of the four loci were linked to each other, 16 genotypes appear in the F_2_ generation in each cross, which could be classified into eight and three phenotypes in Crosses I and II, respectively (Table 3). The *b* and *r* loci were phenotyped by intact observations of the black melanophores and orange xanthophores in the skin. To phenotype SLa expression, we genotyped the *ci* and *SLa* loci by PCR-RFLP using the primer set F: 5′-CAGCTCCAAAAGTGAAATCCAAC and R: 5′-TGCACAGTTGTATTTGTCAGTTTG. These primers were designed at exons of the *ci* locus to sandwich the frame-shift *ci* mutation and an intron. Therefore, if genomic PCR amplifies a short intron-less product, the fish has the *SLa*-expressing cDNA construct (the *SLa*^*+*^ allele) and overexpresses SLa (SLa++). If the primers amplify only a longer product with the intron that could be cut by NspI, the fish has the wild-type *Ci* allele and expresses SLa at the wild-type level (SLa+). If the longer product could not be cut by NspI, the fish is homozygous of the mutated *ci* allele and does not express SLa (SLa–).

### Measurement of hepatic triglycerides

We anesthetized adult fish on ice, pithed the brain, and isolated the liver from the abdominal cavity using fine scissors and forceps. In our previous experiments [2,13], the livers taken out from the cavity were directly used for lipid extraction. We first followed this method, but soon were confronted with a problem that percent hepatic triglycerides often vary over tenfold, even between fish of the same strain kept in the same tank. We assumed that oil-like droplets attaching to the liver, which could only be detected by binocular-microscopic observation, could be the cause and modified the protocol to remove all the droplets (and nonhepatic tissues) using fine forceps in Ringer’s solution [38] before using the liver for lipid extraction. As expected, this improvement significantly decreased the coefficient of variation (CV) of the percent hepatic triglycerides (Table 1; *P* = 0.037, paired two-tailed *t* test) and we added this step in all the following experiments. The trimmed liver was put into a microcentrifuge tube with a known weight, and we absorbed the Ringer’s solution using a paper string and measured the weight using an electronic balance.

Lipids in the liver were extracted following the method proposed by Bligh and Dyer [39]. Briefly, we homogenized the liver using a pestle in 0.1 M acetic acid, added methanol and chloroform, mixed well, collected the lower (chloroform) layer, and let it dry in a vacuum evaporator. The lipids remaining at the bottom of the microcentrifuge tube were dissolved into isopropanol. Triglycerides were quantified using a Triglyceride E-test kit (Wako Pure Chemical Industries) and a Nanodrop 1000 spectrophotometer (Thermo Scientific).

### Statistical analyses

Table 1: We calculated the coefficient of variation (CV) for the data and performed a paired two-tailed *t* test to evaluate the difference between before and after the protocol improvement (n = 3). For Actb-SLa:GFP, we used the data of “After 1”.

Table 2: we performed a two-tailed multiple *t* test with Bonferroni correction. Since we repeated the *t* test five times (for body weight, liver weight, HSI, total hepatic triglycerides, and percent hepatic triglycerides), we used *P* < 0.01 to guarantee the significance of *P* < 0.05.

Figure1: We analyzed all of the data using a one-way ANOVA and Tukey’s *post hoc* HSD test.

Figure2: The data were classified according to either the environmental conditions (control, black background, white background, and fasting; n = 6 for each; Figures 2a and 2b) or the genetic conditions (*ci*, Actb-SLa:GFP, and HNI; n = 8 for each; Figures 2c and 2d), and analyzed using a two-way ANOVA. We used a Dunnett *post hoc* test for the environmental comparison regarding the “control” as a reference group and Turkey’s *post hoc* HSD test for the genetic comparison.

Figures 3a and 3b: The data from the Cross I siblings were classified into two groups based on any of the four phenotypes (sex, the level of SLa expression, melanin synthesis, or xanthophore differentiation). We could not determine one of the four phenotypes in 21 siblings (Table 4). Instead of ignoring all of these siblings, we analyzed the data using a two-tailed multiple *t* test with Bonferroni correction. Because we repeated the test eight times in total, we adopted *P* < 0.00625 to guarantee *P* < 0.05.

Figure3c: The data from the Cross II siblings were classified into three groups according to the SLa expression. The data were analyzed using a one-way ANOVA and Tukey’s *post hoc* HSD test.

## Authors’ contributions

Y.S. and A.Y. performed all the physiological and genetic experiments and collected the data. S.F. designed the experiments, crossed the fish, analyzed the data, and wrote the manuscript. All authors read and approved the final manuscript.
